# Cluster headache: an update on clinical features, epidemiology, pathophysiology, diagnosis, and treatment

**DOI:** 10.3389/fpain.2024.1373528

**Published:** 2024-03-08

**Authors:** Daniel San-Juan, Karina Velez-Jimenez, Jan Hoffmann, Adriana Patricia Martínez-Mayorga, Agustín Melo-Carrillo, Ildefonso Rodríguez-Leyva, Silvia García, Miguel Ángel Collado-Ortiz, Erwin Chiquete, Manuel Gudiño-Castelazo, Humberto Juárez-Jimenez, Marco Martínez-Gurrola, Alejandro Marfil, Juan Alberto Nader-Kawachi, Paul David Uribe-Jaimes, Rubén Darío-Vargas, Jorge Villareal-Careaga

**Affiliations:** ^1^ Epilepsy Clinic, Instituto Nacional de Neurología y Neurocirugía Manuel Velasco Suárez, Mexico City, Mexico; ^2^ Department of Neurology, Hospital Angeles Lomas, Mexico City, Mexico; ^3^ Institute of Psychiatry, Psychology & Neuroscience, King’s College London, London, United Kingdom; ^4^ Department of Neurophysiology, Universidad Autónoma de San Luis Potosí, San Luis Potosí, México; ^5^ Department of Anesthesia, Critical Care, and Pain Medicine, Beth Israel Deaconess Medical Center, Harvard Medical School, Boston, MA, United States; ^6^ Department of Neurology, Hospital Central “Dr. Ignacio Morones Prieto”, and Faculty of Medicine, Universidad Autonoma de San Luis Potosí, San Luis Potosí, Mexico; ^7^ Clinical Research Department, Centro Médico Nacional “20 de Noviembre”, ISSSTE, Mexico City, Mexico; ^8^ Neurological Center, ABC Medical Center, Mexico City, Mexico; ^9^ Department of Neurology and Psychiatry, Instituto Nacional de Ciencias Médicas y Nutrición Salvador Zubirán, Mexico City, Mexico; ^10^ Star Medica Group, Star Medica Hospital Lomas Verdes, Mexico City, Mexico; ^11^ Department of Neurology, Médica Sur, Mexico City, Mexico; ^12^ Department of Neurology, General Hospital 450, Durango, Mexico; ^13^ Headache and Chronic Pain Clinic, Neurology Service, Hospital Universitario “Dr. J. E. González” of the Universidad Autónoma de Nuevo León, Monterrey, Mexico; ^14^ Neurology and Neurosurgery Center, Médica Sur, Mexico City, Mexico; ^15^ Neurology Center, ABC Medical Center, Mexico City, Mexico; ^16^ Department of Neurology and Psychiatry, Clínica de Mérida, Merida, Mexico; ^17^ Department of Neurology, Hospital General de Culiacán, Culiacán, Mexico

**Keywords:** cluster headache, diagnosis, differential diagnosis, epidemiology, risk factors

## Abstract

Cluster headache (CH) is one of the worst primary headaches that remain underdiagnosed and inappropriately treated. There are recent advances in the understanding of this disease and available treatments. This paper aims to review CH's recent clinical and pathophysiological findings, diagnosis, and treatment. We performed a narrative literature review on the socio-demographics, clinical presentations, pathophysiological findings, and diagnosis and treatment of CH. CH affects 0.1% of the population with an incidence of 2.07–9.8/100,00 person-years-habitants, a mean prevalence of 53/100,000 inhabitants (3–150/100,000 inhabitants). The male-to-female ratio remains inconclusive, as the ratio of 4.3:1 has recently been modified to 1.3–2.6, possibly due to previous misdiagnosis in women. Episodic presentation is the most frequent (80%). It is a polygenetic and multifactorial entity that involves dysfunction of the trigeminovascular system, the trigeminal autonomic reflex, and the hypothalamic networks. An MRI of the brain is mandatory to exclude secondary etiologies. There are effective and safe pharmacological treatments oxygen, sphenopalatine, and great occipital nerve block, with the heterogeneity of clinical trial designs for patients with CH divided into acute, transitional, or bridge treatment (prednisone) and preventive interventions. In conclusion, CH remains underdiagnosed, mainly due to a lack of awareness within the medical community, frequently causing a long delay in reaching a final diagnosis. Recent advances in understanding the principal risk factors and underlying pathophysiology exist. There are new therapeutic possibilities that are effective for CH. Indeed, a better understanding of this challenging pathology will continue to be a subject of research, study, and discoveries in its diagnostic and therapeutic approach.

## Introduction

Cluster headache (CH) is a primary headache belonging to the trigeminal autonomic cephalalgias (TAC) group. The earliest descriptions date back to 1641, when the Dutch physician Nicolaes Tulp, famous for Rembrandt's painting, “The Anatomy Lesson,” described a recurrent intense unilateral headache no longer than 2 h. In the Medical Observations (1), the autonomic features were characterized by Wilfred Harris (1869–1960), a London neurologist, in his classic monograph Neuritis and Neuralgia in 1926 (2); this was the first recognition of CH as a separate entity from migraine and trigeminal neuralgia (2). In 1936, Harris named these headaches migrainous neuralgia or ciliary (migrainous) neuralgia (3), where he reported the one-sidedness of the attacks, the severity, associated autonomic features, and the frequency of attacks. His description was the first recorded report of CH in the English medical literature. The same clinical features are detailed in the International Classification of Headache Disorders-3 (ICHD-3) (4).

CH has the legendary reputation of being the most severe primary headache and one of the most excruciating pain conditions a human being can experience (with terrible intensity and generating the most remarkable restlessness). It is colloquially known as the “suicide headache” as many patients may contemplate suicide during attacks. The lack of education of emergency room physicians and various specialists who are not specifically trained and experienced in the management of headache disorders leads to underdiagnosed and often inadequate treatment of CH. The simplicity of its clinical picture contrasts with the fact that the diagnosis is made with an average delay of 5 years from the first occurrence of attacks, and the correct therapeutic management is provided only to a minority of these patients (5, 6). As a natural consequence of the severity of CH, the disability it causes, and the described deficiencies in health care, patients with CH show a high proportion of sickness absence. Beyond the severe impact on the quality of life of affected CH patients, the financial benefits resulting from sick leave or statutory sick pay and the higher number of disability pensions cause a significant cost to society (5, 6).

## Definition

TACs are primary headache disorders characterized by pain localized in the first division of the trigeminal nerve in parallel with ipsilateral cranial autonomic features. CH is the most common and best-studied TAC. It is characterized by severe pain attacks, strictly unilateral, of orbital, supraorbital, temporal, or any combination of these locations, the average duration ranging from 15 to 180 min and occurring from once every two days to eight times a day when in the active phase. Attacks accompany ipsilateral cranial autonomic symptoms such as lacrimation, ptosis, ocular flushing, rhinorrhea/nasal congestion, miosis, and restlessness. Most patients suffer from episodic CH with attacks occurring in episodes (i.e., clusters), usually lasting weeks to months, separated by attack-free periods, which can last between three months to several years. About 10%–20% of patients have a chronic variant without significant attack-free periods (less than three months/year). Attacks usually follow a circadian pattern, commonly occurring around the same time of the day. As attacks naturally also happen at night, they significantly impact sleep quality, leading to several consequences and adding to the disease burden (4).

The clinical picture of CH and its diagnostic criteria are defined in the current version of the International Classification of Headache Disorders (ICHD-3) published by the International Headache Society (IHS) (4).

## Epidemiology

CH affects 0.1% of the population (7) and seems more frequent in males, but there is contradictory data on this aspect. Previously, it was thought that CH mainly affects men. Still, the difference in the prevalence between sexes is decreasing, not because the prevalence is changing, but because more women are being correctly diagnosed. A typical feature of CH is the circannual variation in its incidence (more frequent in the spring and the autumn) (8). Fifty-five percent of patients with CH have suicidal ideation, although it is rare for them to commit it (4), and depression occurs almost three times more often than in controls (8). Delayed diagnosis in young people is also common (9).

### Incidence

The incidence has been difficult to estimate due to the relatively low frequency of CH and systematic underdiagnosis. A study in a specialist practice setting in the USA observed 40 new cases of TAC in 4 years, mostly CH, which accounted for 5.3% of all headaches (10). An investigation in Olmsted, Minnesota, found an overall age- and sex-adjusted incidence from 1979 to 1981 of 9.8/100,000 person-years and from 1989 to 1990 of 2.07/100,000 person-years (11). Part of the problem already starts at universities where headache disorders do not play a significant role in teaching (in fact, they are almost non-existent). In contrast, the other problem is a general perception that “it is only a primary headache.”

### Prevalence

Fischera et al. (7) reported in a meta-analysis of 16 studies that examined prevalence frequencies from 3 to 150/100,000 persons, and the combined lifetime prevalence was 124/100,000 (95% CI: 101–151), and the mean annual prevalence was 53/100,000 (95% CI: 26–95). CH is considered a disease of age-productive males, with a mean male-to-female ratio of 3:1 (12); this ratio was reported for many years, but this ratio has recently been modified with a reduction of the masculine predominance to 6.2 (13) to 1.47 (14), possibly due to previous misdiagnosis in women where CH may be mistaken for migraine. Possibly, there may not be a considerable male predominance (7). The lifetime prevalence of CH is stable: approximately 1 in 1,000 persons suffer from CH, and the prevalence is independent of region and population (15).

Regarding the evolution and prognosis of episodic CH, 80.7% of patients will remain in the episodic form, whereas 12.9% will evolve to a chronic condition, and 6.4% will be in both presentation forms within ten years. Of the 12.9% of cases with chronic CH, 52.4% will remain chronic, 32.6% will revert to episodic, and 14.3% will develop both forms. Poor prognosis is related to the older age of onset, being male, and more than 20 years duration for episodic presentation (16).

Variations can be found in epidemiological data from different series; Stovner et al. (16) determined that Multiple Linear Regression analyses explained less than 30% of the variations; other methodological factors influencing these differences are year of publication, sample size, the inclusion of probable diagnoses, sampling method, screening question, and research setting (17). Table 1 summarizes the results of prevalence studies worldwide from 2002 to 2023.

**Table 1:** Cluster headache prevalence studies in various research studies worldwide from 2002 to 2023.

Country/year of publication	Sample	Affected according to HIS criteria (male/female)	Ratio male: female	Episodic/chronic	Prevalence reported	References
Norway/2003	1,838	7 (6/1)	6:1	6/1	381 Annual prevalence per 100,000	(18)
Taiwan/2004	10,934 registers of Headache patients	104 (90/14)	6.4:1	104/0	103 Annual prevalence per 100,000	(19)
Italy/2005	7,522	21 (12/9)	1.3:1	17/4	279 Annual prevalence per 100,000	(20)
Sweden/2006	31,750 twins born from 1935 to 1958	45 (37/8)	4.6:1	39/6	1 per 500 of the general population	(21)
Germany/2007	3,336	4 (3/1)	3:1	4/0	119/Annual prevalence per 100,000	(22)
Germany/2007	1,312	2 (2/0)		2/0	120,000 Cases of CH in Germany in 2005.	(23)
Georgia/2009	1,145	1 (1/0)		1/0	lifetime prevalence of CH 87 per 100,000	(24)
China/2013	16 regions of China	120 (105/15)	7:1	111/9	Without data	(25)
Ethiopia/2013	231 patients with primary headache	3 (3/0)		Without data	1.3% of the sample	(26)
India/2014	Without data	33 (30/3)	10:1	33/0	Without data	(27)
Korea/2017	Data base of 7 headache clinics	200 (175/25)	7:1	199/1	Without data	(28)
Brazil/2018	36,145	15 (13/2)	6.5:1	Without data	0.0414%	(29)
USA/2021	3,251	79 (54/25)	2.1:1	75/4	2.4% of the sample	(30)
Iran/2022	570 patients of ≥50 of headache clinic	24 (13/11)	1.18:1	Without data	4.2% of the sample	(31)
Norway/2022	3,892,260	1,891/(1,126/765)	1.47:1	Without data	48.6 per 100,000	(14)
Japan/2023	11,842	420 (336/74)	4.5:1	401/19	3.5	(32)
Sweden/2023	1,484 patients of cluster headache biobank	874 (575/299)	1.9:1	719/175	Without date	(33)

## Risk factors

### Familial factors

CH is considered a polygenic and genetic-environmental multifactorial disorder. Positive family history varies from 0% to 22% (median 8.2); in monozygotic twins, the concordance is 5.4%. There are families where a probable autosomal dominant or recessive transmission of low penetrance has been postulated (10), with no causal candidate genes to date. Individuals with first-degree relatives with CH are 5–18 times more likely to experience CH than the general population, and if the relative is a second-degree family member, the risk is 1–3 times higher (34). In a meta-analysis, O'Connor et al. observed that the estimated true prevalence of family history was 6.27% (95% CI: 4.65–8.40%). In the sex-adjusted model, the familial prevalence was 9.26% (95% CI: 6.29–13.43%) in women (35).

### Sex

CH used to be more frequent among males, with a mean ratio of 6:1; however, in chronic forms and among people aged 20–49, this is as high as 11:1 (36). However, with better knowledge of the disease, exciting epidemiological changes have been reported. It usually shares a similar clinical profile in both sexes, except for nausea, which is more marked in women (37), who present two peaks of higher frequency in the second and sixth decades of life (38). There are proposals to explain this, such as more accurate and timely diagnoses and changes in lifestyle (smoking and alcohol use, which is important now also in women) (6, 39).

### Age of onset

The mean age of onset varies, although on average, it is 30.2 ± 13.8 years (30.1 ± 13.0 in men and 30.4 ± 15.7 in women). Women with chronic CH have an age of onset at 42.8 ± 21.7 years, although women with secondary CH did not differ much from those with episodic CH (40).

### Smoking

Smoking history is approximately 60% associated with an earlier onset of CH, an increased male/female ratio, and a lower response to triptans (39). Illicit drug use is higher in the Dutch CH population than in the general population (31.7% vs. 23.8%; *p* < 0.01). Both associations, tobacco and illicit drug use [such as psilocybin mushrooms, lysergic acid diethylamide (LSD), heroin, amphetamine, and cannabis], may be due to a shared factor between CH and addictive behavior (41).

### Traumatic brain injury

An antecedent of traumatic brain injury (TBI) is frequent but not necessarily related. However, there is usually no temporal relationship between the head trauma and the onset of CH, in addition to not meeting the criteria for posttraumatic headache. However, a cohort included 553 patients with primary CH, identifying 26 patients with episodic cluster headache (ECH) with the antecedent of TBI. Multivariate analysis revealed significant associations between post-traumatic headache with cluster headache phenotype (PTH-CH) and family history of CH (OR: 3.32, 95% CI: 1.31–8.63), in the chronic form (OR: 3.29, 95% CI: 1.70–6.49). Patients with PTH-CH were at higher risk of being intractable to acute (OR: 12.34; 95% CI: 2.51–64.73) and preventive treatments (OR: 16.98; 95% CI: 6.88–45.52) and associated chronic migraine (OR: 10.35; 95% CI: 3.96–28.82) (42).

### Population

Available research findings are mainly based on the studies conducted in the Caucasian population (43). A study in the USA observed that about 25% of African American women suffered from CH compared to 17.4% of men of the same group (6). The phenotype of CH has been reported to differ among East Asians, who have less agitation and restlessness and a lower prevalence of chronic forms (44). In a retrospective cohort study involving patients attending seven tertiary Headache Centers in Italy, out of twenty-eight thousand eighty-three patients, “rare headaches” were recorded in 822 (4.1%) prevalent cases and 461 (2.3%) new cases. Cluster headache is the most frequently diagnosed rare headache (70.4%), of which 59% is episodic and 11% is chronic (45). Although trigeminal and autonomic headaches are rare, they must be recognized, especially cluster headaches, because of their impact on quality of life (6).

## Impact on quality of life and burden of disease

A Danish study documented the personal and occupational limitations of 400 patients with CH, estimating that 94% of them had restrictions during pain attacks. Patients rated their health as poor/very poor in the episodic form in 9% compared to 1% of controls. In the chronic presentation, the odds of rating health as good/very good were ten times lower (OR: 10 < 10, 95% CI: 5.29–18.79). The odds of receiving a disability pension were five times higher in the chronic compared to the episodic form (OR: 5.0, 95% CI: 2.3–10.9, *p* < 0.001). The individuals who were in presenteeism from their employment despite having a CH attack are estimated to have a 65% reduction in their productivity (46).

## Pathophysiology

The pathophysiology of CH still needs to be understood. The current understanding is based on preclinical, clinical, and imaging studies in patients with the disease. However, research in CH needs to catch up to other primary disorders, such as migraine, as animal models remain scarce, and clinical research is hampered by the severity and short duration of attacks and attack bouts, which complicate the recruitment of study subjects. Finally, because of the relatively low prevalence compared to migraine, funding for CH research remains very limited and is mainly directed to other more prevalent primary headache disorders, such as migraine (6).

To better understand the changes that occur in the clinical picture of CH, nitric oxide (NO) and its prodrug nitroglycerin have been used to provoke migraine-like attacks in patients previously diagnosed with migraine and cluster-like attacks in patients which have been diagnosed with cluster headaches and are in the bout. Nitroglycerin increases CGRP levels by causing vasodilatation and hyperactivity of trigeminal nociceptive fibers, as demonstrated by Fanciullacci in his studies (47, 48). The same occurs following the administration of CGRP (49, 50). Whether the effect of NO is via CGRP or if the impact attack-triggering effects are independent of each other has yet to be clarified as the available data are inconsistent. However, NO and CGRP can most likely trigger attacks by separate mechanisms, although the two may interact with each other. Other inducers of CH attacks can include, besides CGRP, PACAP38, and VIP, which can act on the mast cell (15, 51–53).

**Figure 1:**
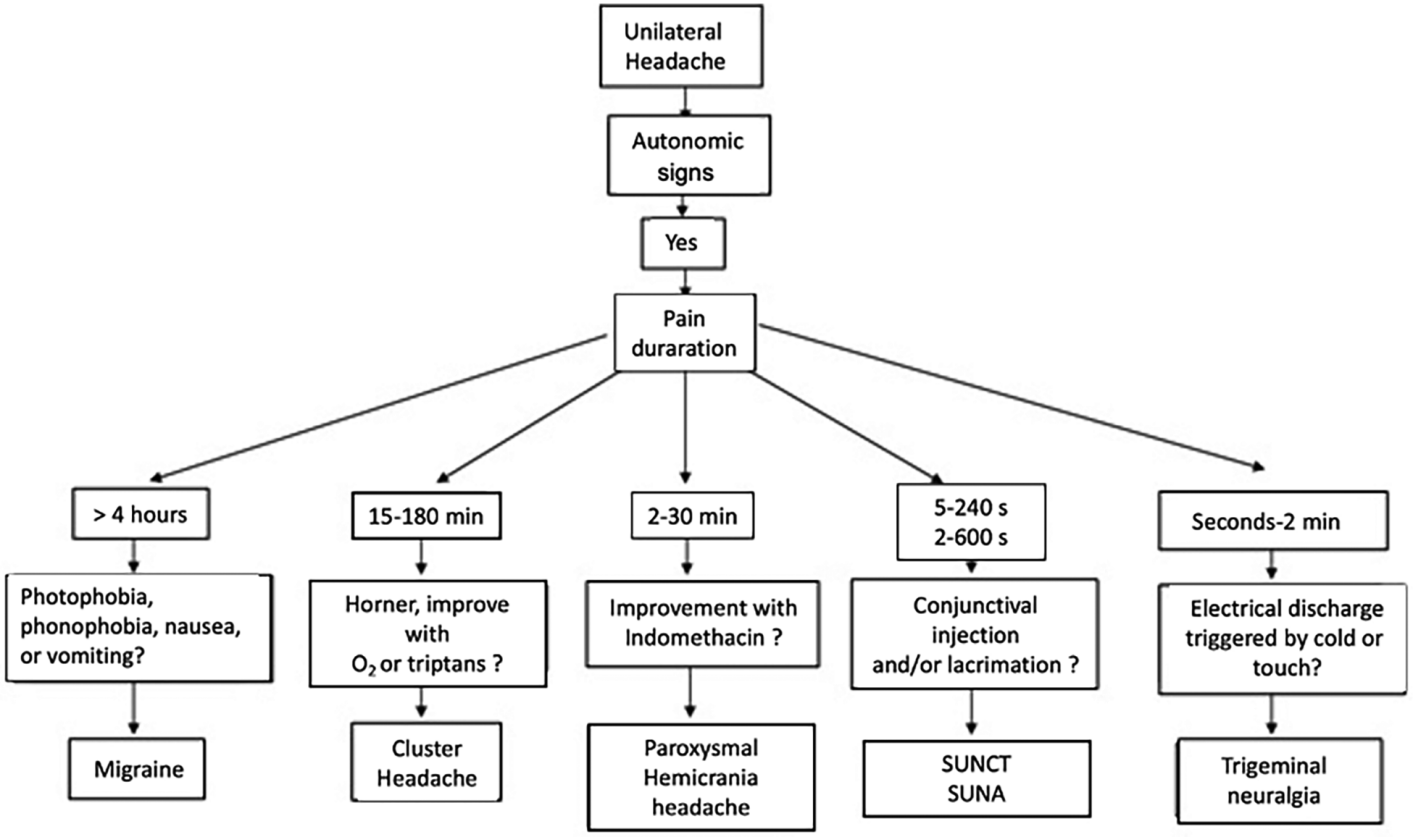
Differential diagnostic algorithm for cluster headache.

**Figure 2:**
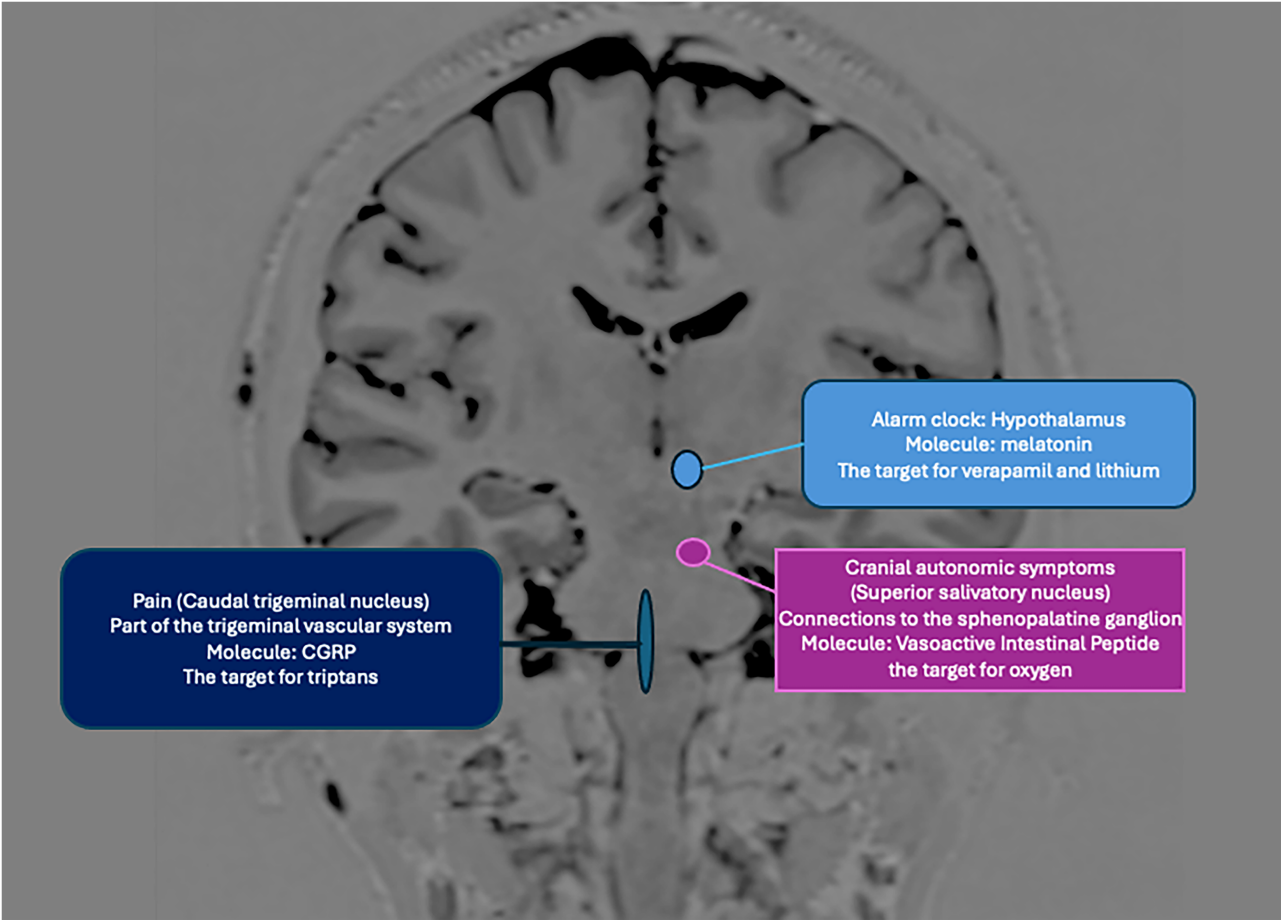
The anatomical components and neurotransmitters involved in CH involve hypothalamic control, mainly its suprachiasmatic nucleus, which acts on the superior salivary nucleus (parasympathetic system), the trigeminal-vascular complex. Nociceptive activation is generated in the peripheral nervous system (CGRP) but also centrally, resulting in a parasympathetic outflow (lacrimation, conjunctival injection) facilitated by a sympathetic deficit (miosis, ptosis) inherent to the crisis, in addition to CGRP, the vasoactive intestinal peptide, melatonin, and others (acetylcholine, serotonin, neuropeptide Y) are involved. Verapamil, lithium, triptans, and oxygen use different structures and molecules as targets in their action (54, 55).

**Figure 3:**
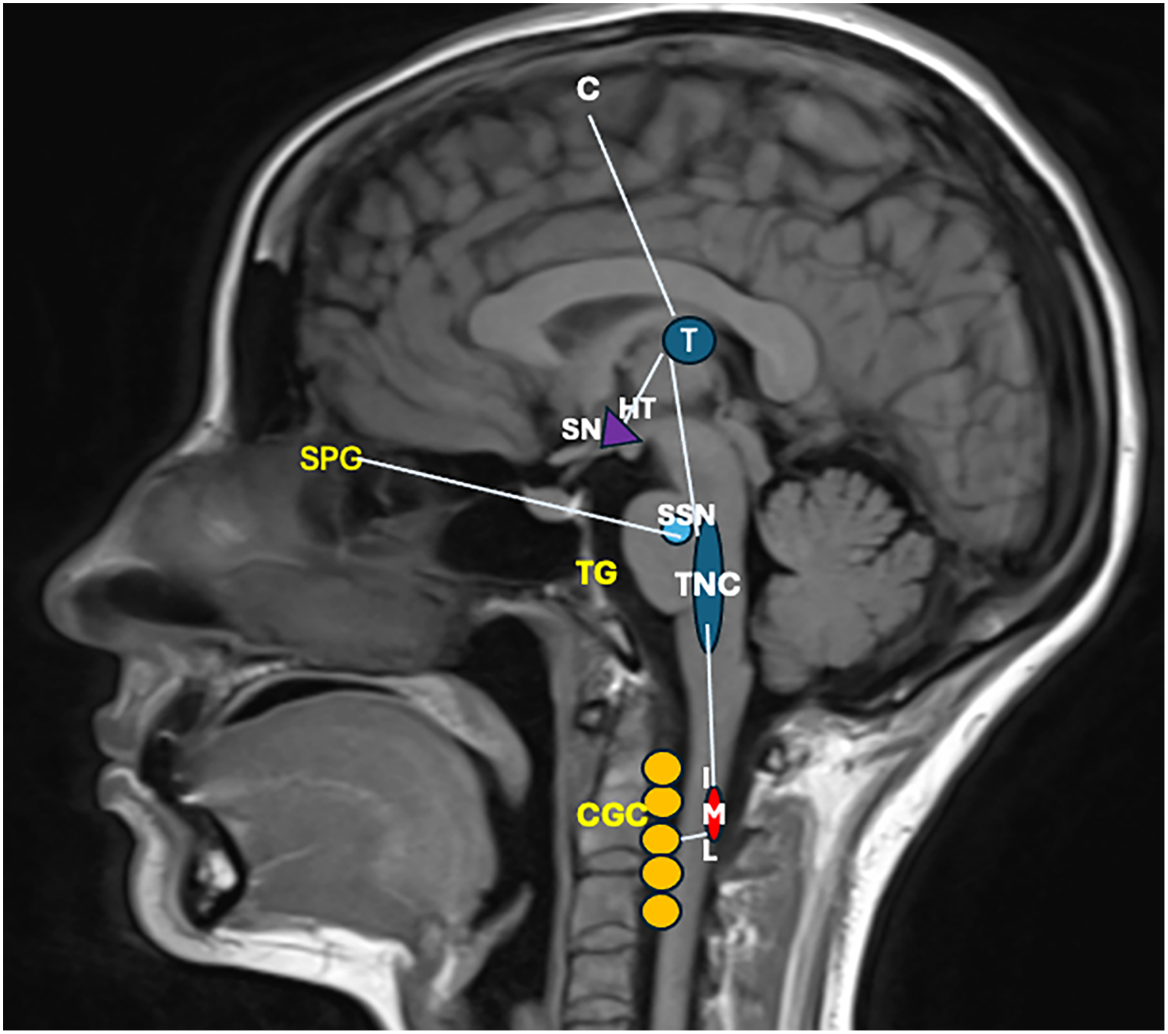
The pain afferents come from the ophthalmic branch of the trigeminal nerve, whose neuronal body is in the trigeminal ganglion (TG), with vascular and dura mater signals; the information enters the trigeminal caudal nucleus (TNC), which carries nociceptive information to the ventral posteromedial nucleus of the thalamus (T) and the primary sensory cortex the information is perceived as pain (C) the information also reaches the hypothalamus (HT), activating the superior salivatory pontine nucleus (SSN) and causing a vasodilatory response. Within the hypothalamus, the suprachiasmatic nucleus is also involved in the pain sufferer's circadian function and alarm clock. In addition, the parasympathetic activation of the sphenopalatine ganglion (SPG) favors patients’ tearing. Concomitantly, the sympathetic system is activated; from the HT, the information descends to the intermediolateral nucleus of the lateral horn of the cervical spinal cord (IML) and from there to the cervical ganglionic complex (CGC), which explains the Horner's syndrome of some patients (ptosis, miosis) (6). A GWAS study compared 852 CH cases from the UK and 591 from Sweden with 5,614 and 1,134 controls, respectively, identified a locus on chromosome 1 and confirmed a previous locus in the UK analysis on chromosome 6, which overlaps with a migraine locus. The major single nucleotide polymorphisms were rs113658130 (p = 1.92 10 17, odds ratio [OR] = 1.51; 95% confidence interval [CI] = 1.37–1.66) and rs4519530(p = 6.98 10 17, OR = 1.47; 95% CI = 1.34–1.61) on chromosome 2, rs12121134 on chromosome 1 (p = 1.66 10 8, OR = 1.36; 95% CI = 1.22–1.52), and rs11153082 (p = 1.85 10 8, OR = 1.30; 95% CI = 1.19–1.42) on chromosome 6. These results have immunologic and pathogenic implications in CH (56).

The neuroanatomical and functional systems involved in the pathophysiology of CH can be divided into three principal components: (1) the trigeminovascular system, (2) the trigeminal-autonomic reflex (sphenopalatine ganglion stimulation), and (3) the hypothalamic system (15). The interaction of these three components is responsible for the characteristic clinical presentation of CH. We will discuss these components in detail in Figures 1–3.

### Trigeminovascular system

This system is formed by neurons innervating the dura mater and meningeal vessels, whose neuronal body is in the trigeminal ganglion. These are pseudounipolar neurons and synapse with neurons of the trigeminal-cervical complex, which are composed of the trigeminal nucleus in the caudal part of the brainstem, as well as the C1 and C2 regions of the spinal nerves (57). These second-order neurons send projections to the thalamus, activating pain-related cortical structures such as the prefrontal cortex, insula, and cingulate cortex (58). Activating the trigeminovascular system releases neuropeptides at trigeminal nerve endings; these peptides include CGRP, substance P, and neurokinin A, among others. Due to trigeminal and cervical innervation, the clinical presentation of CH suggests the activation of second-order neurons as responsible for the perceived pain in patients (57, 59–60).

### Trigeminal-autonomic reflex

CH attacks involve activation of parasympathetic outflow, which causes typical trigeminal-autonomic symptoms such as lacrimation, conjunctival injection, and nasal congestion. In the context of TACs, the trigeminal and parasympathetic systems interact by a so-called trigeminal-autonomic reflex. The pathway of this reflex starts with the activation of the second-order neurons of the trigeminovascular system located in the cervical complex (trigeminal nucleus, C1, and C2); these neurons send projections to the parasympathetic system through the superior salivary nucleus situated in the pons. These projections travel through the facial nerve and synapse in the sphenopalatine ganglion. Postganglionic parasympathetic nerves innervate the lacrimal, nasal, and pharyngeal glands (61, 62). These neurons contain NO-synthase, vasoactive intestinal peptide (VIP), CGRP, and the Pituitary Adenylate Cyclase-Activating Peptide-38 (PACAP-38). The exact mechanism of the activation of this reflex remains unknown (63–65).

### Hypothalamus

The hypothalamus is a structure that plays a vital role in regulating circadian rhythm, neuroendocrine homeostasis, and the autonomic nervous system. The hypothalamus also plays a role in the nociceptive process of the trigeminovascular system and receives projections from the trigeminal nerve via the trigeminal-hypothalamic tract (66–69). The mechanistic relevance of the hypothalamus in CH is supported by circannual patterns, attack phenotypes, and accompanying neuroendocrine hormonal alterations, as well as by several neuroimaging studies that revealed an activation of the posterior hypothalamic region during attacks of CH (70–72). Pre-clinical studies have shown that neuropeptides such as orexins, somatostatin, GABA, and 5-HT receptors in the paraventricular region of the hypothalamus can modulate nociceptive neurotransmission (73–79).

Neuroanatomical connections of the hypothalamus suggest that it may also contribute to the autonomic symptoms of CH. The paraventricular region of the hypothalamus has direct projections to the superior salivary nucleus, which in turn projects to the sphenopalatine ganglion and facial nerve, lacrimal, nasal, and pharyngeal glands (58). Stimulation of the superior salivatory nucleus generates increased blood flow to the lacrimal glands, which may explain some symptoms. The suprachiasmatic nucleus of the hypothalamus is the principal circadian pacemaker, and perturbations of the mechanisms regulating this nucleus may contribute to CH (67–70). The suprachiasmatic nucleus can be affected by photoperiodism (changes in sunlight duration during the day), which is strongly associated with increased CH attacks. In addition, the volume of the suprachiasmatic nucleus changes seasonally, being twice as long during autumn and summer. These observations suggest a relationship between the onset of headache attacks and photoperiodism (71–77).

Finally, studies using positron emission tomography have demonstrated activation of the hypothalamus during CH attacks, mainly posterior ipsilateral activation of the hypothalamus, as well as the so-called pain matrix (prefrontal cortex, thalamus, cingulate cortex, insula, and cerebellum) (78).

The trigeminovascular system and the trigeminal-autonomic reflex not only modulate each other but also can potentiate each other through the release of vasoactive neuropeptides. The activation of the trigeminal-autonomic reflex may be secondary to an activation of the trigeminovascular system; however, peripheral activation of afferent and efferent trigeminal-autonomic reflex branches is insufficient to generate CH attacks (10). The hypothalamus likely plays a central role in the pathophysiology, particularly in creating a brain state in which attacks are made possible. This may explain why cluster-like attacks can only be triggered with CGRP (which does not cross the blood-brain barrier) while CH patients are in the bout (75). However, more information is needed to understand the specific role of each hypothalamic nucleus in CH, its function in the induction of autonomic symptoms, and its photoperiodicity.

## Differential diagnosis

The diagnosis of CH is primarily clinical, and neuroimaging studies are indicated in specific cases to rule out secondary headaches (4). Frequently, there is a significant delay in diagnosing CH, which is essential when planning optimal medical management. In this context, Byung-Su Kim et al. showed in a multicenter registry in South Korea with a 4-year follow-up period that in 36.4% of patients, the average diagnostic delay was 5.7 ± 6.7 years (77). A Danish study reported that the leading causes of diagnostic delay included prolonged attack duration (greater than 180 min), migraine-like clinical features, and predominantly nocturnal episodes (75). A survey on CH conducted in the United States revealed that 21% of patients received an adequate diagnosis at the onset of the problem. There could be an average delay of 5 years after the initial start of the CH (76).

When CH is misdiagnosed, the clinical picture is most confounded as migraine, either because the clinical picture is misinterpreted or because both headache disorders occur as comorbidity, which is then not recognized. Other common misdiagnoses include trigeminal neuralgia, sinusitis, or dental and jaw disorders, leading to unnecessary treatments and procedures and increased anxiety, depressive disorders, and even suicidal ideation (79, 80). The Erwin test is a tool that identifies patients with CH. This test consists of 3 questions: Is this the worst pain you have experienced? Does your pain last less than 4 h? Do one or more of the following symptoms or signs occur during the headache: unilateral red eye, unilateral lacrimation, unilateral rhinorrhea, or unilateral nasal congestion? In the case of an affirmative response to all three questions, a sensitivity of 85% and specificity of 89% of correct CH diagnosis is obtained (81), proving to be a valuable and easy-to-use tool for proper diagnosis.

Differential diagnosis can be challenging. Nevertheless, it should be guided by anamnesis and initial physical examination, followed by laboratory studies to determine if there is any other etiology of the paroxysmal neurological events, e.g., serum electrolyte disturbances or hepatic or renal insufficiency, lumbar punctures in case of suspected neuro infection. Neuroimaging studies such as brain tomography or magnetic resonance imaging (MRI) are indicated in patients with an atypical clinical picture to rule out other secondary etiology because of specific vascular alterations that can be found in 37.7% (e.g., internal carotid artery dissections, arteriovenous malformations of any cerebral lobe, intracranial aneurysms [vertebral artery, inferior cerebellar artery, posterior communicating artery, multiple, internal carotid], artery dissection, intracavernous internal carotid artery thrombosis, dural arteriovenous fistulas, and cerebral venous thromboses), tumors in 32.5% (prolactinomas, epidermoid cysts, meningiomas, parietal glioma, besides others.) and inflammatory entities in 27.3% (sinusitis of any sinus, sphenoid sinus mucocele, inflammatory orbital pseudotumor, posterior scleritis, idiopathic orbital myosis, hypothalamic-pituitary granuloma, and idiopathic intracranial hypertension). These lesions typically occur ipsilateral to the symptoms and can, therefore, mimic CH (81, 82).

For these reasons, it is recommended to perform cranial MRI in patients with suspected CH (82). The American College of Radiology recommends using contrast medium on MRI (83). In patients who do not have symptomatology suggestive of pituitary adenoma, MRI with sella turcica focus is not necessary, as a retrospective study demonstrated that the prevalence of pituitary adenomas in patients with CH is like that reported in the general population (38). Since arterial dissection can simulate a CH, the European Headache Federation has recommended using supra-aortic truncal angio-resonance or carotid and vertebral artery ultrasound in selected patients (83, 84).

In summary, an MRI of the brain with contrast is recommended in every patient with CH, an MRI of the *sella turcica* in suspected pituitary tumors and in patients with suspected cerebrovascular lesions, an angio-MRI or MRI of aortic trunks (83).

Atypical features of CH that should alert the clinician are (1) pain attacks being exclusively ocular or retro-ocular, (2) abnormal findings on neurological examination, (3) other headache attacks between those typical of CH, (4) atypical duration of CH, (5) migraine-like symptoms, (6) Horner's syndrome, and (7) an unexpected frequency of atypical attacks, being the most relevant, in particular cranial nerve disorders, within which ophthalmological signs and symptoms are the most frequent. In all cases of primary CH, the findings of laboratory or neuroimaging examinations are expected to be normal or unrelated to the etiology of CH (84–86).

Table 2 and Figure 1 show the diagnostic approach to CH concerning other common primary headaches.

**Table 2:** The main clinical features of the differential diagnoses of primary headache disorders about CH are shown.

Characteristics	Cluster headache	Migraine	Paroxysmal hemicrania	SUNCT/SUNA	Trigeminal neuralgia
Pain distribution	Orbital, periorbital, or temporomandibular pain. Strictly unilateral	Frontotemporal predominance. Generally unilateral	Orbital, supraorbital, or temporal region.	Orbital, supraorbital temporal region. Strictly unilateral	Trigeminal nerve distribution of predominance in V2 and V3.
Duration of episodes	15–180 min (average 100 min)	More than 4 h (4–72 h)	2–30 min	1–600 s	Sudden (sec to 2 min)
Type of pain	Piercing	Throbbing	Piercing	Stabbing/Saw-tooth	Stabbing, electrical
Intensity	Very intense	Moderate to intense	Intense	Moderate to intense	Intense
Autonomic disturbances	Present in 94% of patients, unilateral	Present in 56%, tend to be bilateral and fluctuating.	Present	Present	Mild lacrimation/redness
Accompanying symptoms	Restlessness, agitation	Nausea, vomiting, photophobia and sonophobia	Restlessness or agitation		In continuous pain structural cause must be ruled out
Worsening with exercise	No	Yes	No	No	No
Triggers	Alcohol, cigarette, nitroglycerin, odors, heat	Stress, sleep deprivation, fasting, menstruation	Alcohol, neck movements	Tactile stimulus, chewing or toothbrushing	Cold, touch
Predominant gender	Male. Ratio 4:1 20–40 years	Female Ratio 3:1	Female 30–40 years old Ratio 2:1	Male 20–60 years old. Ratio 1.5:1	Female Older than 50 years
Temporality pattern	Episodic: Episodes from 7 days to 1 year separated by at least 3 months. Chronic: More than 1 year without remission or remission less than 3 month	Episodic: Less than 15 episodes per month. Chronic: 15 or more days per month for more than 3 months	Episodic: Episodes from 7 days to 1 year separated by at least 3 months. Chronic: More than 1 year without remission or remission less than 3 months.	Episodic: Episodes from 7 days to 1 year with remission of three or more. Chronic: More than 1 year without remission or remission of less than 3 months	Paroxysmal: Recurrent paroxysms, without pain between attacks. Continuous: Continuous or almost continuous pain
Most used drugs	100% O2 12–15 L per min for 20 min/triptans	Nonsteroidal anti-inflammatory drugs/triptans	Indomethacin, minimum adult dose 150 mg	Lidocaine IV 1.3, 5 mg/k/h.	Carbamazepine

SUNCT, short-lasting, Unilateral, Neuralgiform headache attacks with Conjunctival injection and tearing; SUNA, short-lasting, Unilateral Neuralgiform headache with Autonomic symptoms.

Resistance to standard treatments for CH should increase the suspicion of a secondary origin. However, acute treatments with triptans, ergotamine, analgesics, or caffeine can improve attacks by up to 46.9%, so a response to therapy does not justify conducting further investigations (87).

Misdiagnosis of CH has a dramatic impact on the patient's quality of life. A survey in the United States revealed that even if the diagnosis is established correctly, 25% of patients have lost their jobs due to the disease, and 8% are unemployed (76). In addition, as events tend to be more predominant at night, they significantly affect sleep quality, adding to the disease burden. Using the Headache Impact Test-6 (HIT-6) in patients with CH, several authors observed that up to 74% were classified as having a severe impact, with 78% reporting daily restrictions and up to 96% a need for lifestyle changes (88), demonstrating the detriment of the quality of life of these patients.

## Treatment

There are effective and safe pharmacological and non-pharmacological treatments with the heterogeneity of clinical trial designs for patients with CH divided into acute, transitional (short preventive treatment or bridging), and preventive long-term interventions. Acute attacks are treated using triptans, oxygen, and—in the case of episodic cluster headache—non-invasive transcutaneous vagal nerve stimulation. Prednisone is the most studied in the bridging phase. Moreover, verapamil and monoclonal antibodies are considered the first option, followed by multiple pharmacological and non-pharmacological options for preventive treatment (89).

## Acute treatment

During acute attacks of CH, the use of triptans is widely recommended. Sumatriptan 6 mg subcutaneously is one of the most effective acute treatments (90, 91). Alternative options with similar efficacy are sumatriptan 20 mg (intranasal) and zolmitriptan 5/10 mg (intranasal). With an overall response rate of triptans in CH of 80%, triptans remain one of the most effective acute treatment options in CH (92, 93). However, due to their vasoconstrictive properties, they are contraindicated in patients with comorbid cardiovascular pathologies (94). In addition, its use is limited to 2 doses in 24 h, which is problematic for patients who experience more than two attacks in 24 h. Unfortunately, Mexico has no access to intranasal or subcutaneous formulations of triptans, which commonly leads to the use of an oral triptan with an analgesic such as naproxen (or other NSAID) to abort the acute pain (95).

Another accessible and highly effective acute treatment is high-flow oxygen, which can be administered at the patient's home or in the emergency room. A non-rebreather mask is used at a flow of 100% oxygen at a rate of 7–15 L/min. Response rates range from 62% to 100% of patients with acute attacks, with a positive answer in 12–15 min on average (96–98).

Based on the high response rates, intranasal and subcutaneous triptans, and oxygen are considered first-line options for the acute treatment of CH attacks. Additionally, non-invasive transcutaneous vagal nerve stimulation is a non-pharmacological option proven effective in episodic CH, reducing the pain intensity within 15 min of stimulation with a favorable safety profile (99).

## Bridging treatment

In the transitional phase, using prednisone orally at 100 mg/day for five days and gradually decreasing the doses is recommended (100). Another alternative, which is probably similarly effective but better tolerated, is the greater occipital nerve block (101, 102).

## Preventive treatment

Verapamil, a calcium channel blocker, is the first-choice pharmacological treatment for preventing episodic and chronic CH using at least 240 mg/day (103). When using verapamil, it is essential to conduct an electrocardiogram before initiating treatment and after each dose increase to identify potential cardiac side effects. Even after a stable dose has been determined, electrocardiograms should be conducted regularly, as cardiac side effects can appear with a significant delay (104–106). Interestingly, in Mexico and other Latin American populations, verapamil doses of no more than 240 mg/day are used, unlike in different latitudes where the recommended doses are up to 720 mg/day of this drug, which possibly has a genetic explanation (103, 107, 108).

The monoclonal antibody galcanezumab is also an FDA-approved preventive treatment option for CH (300 mg/month for two months). The respective randomized controlled trial showed that it decreased weekly attack frequency by 8.7 compared to 5.2 in the placebo group at week three. The most frequent side effects were nasopharyngitis and pain at the application site (109, 110). Unfortunately, the study on chronic CH did not reach statistical significance in a trial with 237 participants (108). In Mexico, galcanezumab has yet to be approved for use in CH. Besides, galcanezumab does not exist at 300 mg presentation in Mexico. Minor dose regimens have proven successful in some patients, but no data from a randomized clinical trial would confirm these observations (111).

The second line includes lithium, civamide, melatonin, topiramate, sodium valproate, baclofen, gabapentin, and transcutaneous non-invasive vagal nerve stimulation, followed by botulin toxin and sphenopalatine ganglion stimulation as a third line of treatment (9, 54). However, most of these studies have methodological shortcomings, such as, for example, an open-label design or a small sample size. Unfortunately, ATI, the company that made this stimulator, went bankrupt.

## Refractory cluster headache

For refractory CH, the use of invasive treatments such as sphenopalatine ganglion blockade with radiofrequency ablation or neuromodulation may be an option in these patients as in one case series it led to a decrease of pain in 31% at six months of follow-up after radiofrequency treatment or 75% at 24 months of follow-up after neuromodulation (112); however, 81% of patients showed maxillary paresthesia when using neuromodulator treatments or were re-operated (113).

Greater occipital nerve stimulation (ONS) has been successfully used in cases with chronic CH refractory to medical treatment. In the early Fontaine et al. open-label trial, a 77% response rate (improvement >50%) was observed after ONS, with a 68% reduction in frequency and a 49% reduction in severity of CH attacks (114). Therefore, based on the feasibility and costs associated with the different surgical procedures currently available, ONS could be considered the first therapeutic strategy for patients with refractory chronic CH, as shown by the current evidence.

A recent meta-analysis of 45 ONS studies for refractory CH showed a pooled response rate of 57%. Deep brain stimulation (DBS) was the second most studied surgical treatment, with a pooled response rate of 77% (115–45).
